# The interplay between chondrocyte spheroids and mesenchymal stem cells boosts cartilage regeneration within a 3D natural-based hydrogel

**DOI:** 10.1038/s41598-019-51070-7

**Published:** 2019-10-10

**Authors:** Annachiara Scalzone, Ana M. Ferreira, Chiara Tonda-Turo, Gianluca Ciardelli, Kenny Dalgarno, Piergiorgio Gentile

**Affiliations:** 1 School of Engineering, Newcastle University, Claremont Road, Newcastle upon Tyne NE1 7RU United Kingdom; 2 Department of Mechanical and Aerospace Engineering (DIMEAS), Politecnico di Torino Corso Duca degli Abruzzi 29, Turin, 10129 Italy

**Keywords:** Biomedical engineering, Biomaterials - cells

## Abstract

Articular cartilage (AC) lacks the ability to self-repair and cell-based approaches, primarily based on using chondrocytes and mesenchymal stem cells (MSCs), are emerging as effective technology to restore cartilage functionality, because cells synergic functionality may support the maintenance of chondrogenic phenotype and promote extracellular matrix regeneration. This work aims to develop a more physiologically representative co-culture system to investigate the influence of MSCs on the activity of chondrocytes. A thermo-sensitive chitosan-based hydrogel, ionically crosslinked with β–glycerophosphate, is optimised to obtain sol/gel transition at physiological conditions within 5 minutes, high porosity with pores diameter <30 µm, and *in vitro* mechanical integrity with compressive and equilibrium Young’s moduli of 37 kPa and 17 kPa, respectively. Live/dead staining showed that after 1 and 3 days in culture, the encapsulated MSCs into the hydrogels are viable and characterised by round-like morphology. Furthermore chondrocyte spheroids, seeded on top of gels that contained either MSCs or no cells, show that the encapsulated MSCs stimulate chondrocyte activity within a gel co-culture, both in terms of maintaining the coherence of chondrocyte spheroids, leading to a larger quantity of CD44 (by immunofluorescence) and a higher production of collagen and glycosaminoglycans (by histology) compared with the mono-culture.

## Introduction

Articular cartilage (AC) is responsible for providing a load bearing and low friction interface between joint surfaces and is characterised by lack of ability to self-repair^1^. In order to stimulate repair, tissue engineering (TE) strategies combine cells and biomaterials to promote cartilage regeneration *in vitro*, with the cell type and culture environment important in modulating the degree of achievable regeneration of new tissue^2^. The most explored cell sources in this field are autologous chondrocytes and mesenchymal stem cells (MSCs)^3^. While chondrocytes are widely used in cell-based cartilage regenerative approaches, the limited availability of donor sites and low yield of isolated cells from autologous tissue (1–5% of the total tissue volume), remain an issue^4,5^. Oppositely, MSCs are non-haematopoietic and multipotent adult stem cells that can be easily harvested from a number of human tissues^6^. In recent years, MSCs and chondrocytes co-cultures have grown in interest as offering a promising approach in cartilage TE, providing the intercellular signals considered important in regulating cell behaviour^7^. When in co-culture, MSCs secrete trophic or cell–cell communication factors to promote proliferation and to delay de-differentiation of chondrocytes, while chondrocytes can induce chondrogenic differentiation of MSCs in the absence of other stimuli^8^. Co-culture systems are complex and have various mechanisms of action, as direct (cell to cell contact) and indirect (with cell types physically separated) *in vitro* co-culture offering different intercellular interactions. While indirect contact *in vitro* co-cultures have been used to evaluate the effect of cytokines secreted by one cell type on the other via autocrine or paracrine signalling; direct physical contact *in vitro* facilitates cell–cell interactions though surface receptors, enhancing the transduction of the molecular signals coordinating the chondrogenic differentiation (ie. VEGF-164α, TIMP-1, −2 and MMP-13)^9–11^.

The aim of the work presented in this paper was to develop a more physiologically representative co-culture system to investigate the influence of MSCs on the activity of chondrocytes. In order to do this we have encapsulated cells in a natural hydrogel, able to mimic the extracellular milieu which contains physical and chemical cues for cell-driven *in vitro* hyaline-type cartilage tissue development^12^. Smart stimuli-sensitive polymeric hydrogels, including temperature-sensitive and pH sensitive ones, have been widely exploited in regenerative medicine as these do not need any toxic chemical reagents to trigger sol–gel transition^13^. In this work, a thermo-sensitive chitosan (CH) and β-glycerophosphate (BGP) hydrogel, previously explored in different biomedical applications (e.g. local drug delivery and nerve, bone and cartilage tissue engineering) was optimised as an *in vitro* 3D system for MSCs and chondrocytes co-culture^14–16^. Chitosan’s structure and characteristics are similar to those of glycosaminoglycans (GAGs) present in the native cartilage, but it gels slowly^17^. However, the combination of chitosan with BGP, a chemical compound used in the body to transport minerals, allows fast chemical crosslinking such that within 5 minutes a thermally and mechanically stable hydrogel is obtained. The goal of this work has been to use the gel to encapsulate MSCs to prove its cytocompatibility and, then, to seed a chondrocyte spheroid on top of the gel, in order to evaluate if the presence of MSCs could influence the behaviour of the chondrocytes over an extended culture period.

## Results

### Gelation time and thermal irreversibility

Tube inverted method evidenced that CH/BGP system resulted to be liquid at room temperature up to 2h45min ± 10 min, while it became a gel within 5 ± 1 min and 2 ± 0.5 min at 37 °C and 50 °C respectively. The hydrogel thermo-irreversibility was demonstrated maintaining the gel state after keeping the CH/BGP system at 4 °C where it maintained the structural stability without becoming liquid (Fig. S2).

### Water uptake and nutrients diffusion

**Figure 1:**
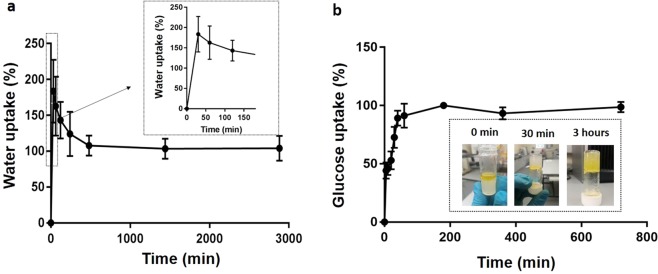
(**a**) Water uptake study of CH/BGP at different time points. (**b**) CH/BGP glucose uptake up to 720 min. Insert: qualitative analysis of nutrient diffusion throughout the hydrogel.

The water uptake ability of the chitosan-based hydrogel at 37 °C is shown in Fig. 1a. The samples displayed an initial rapid water uptake of 184 ± 44% within 30 min. Then, the water uptake over time achieving a value of about 108 ± 14% within 8 h. After stabilisation, a water uptake plateau was observed until 48 h after the immersion of the samples in Dulbecco’s phosphate-buffered saline (DPBS) (101 ± 20%). Moreover, Fig. 1b shows the nutrients diffusion through the hydrogel via absorption of glucose dye solution. Particularly, after 30 min a superficial uptake of the dye from the gel appeared, while after 3 h the gel presented a homogenous yellow colour due to the complete dye uptake. The hydrogel ability to release nutrients over time was evaluated in fluorescence up to 48 h: a rapid 2-(N-(7-Bitrobenz-2-oxa-1,3-diazol-4-yl) Amino)-2-Deoxyglucose (2-NBDG) uptake was found within the first 3 h, reaching a close-to-maximum uptake plateau at 190 ± 10 min.

### Chemical characterisation

**Figure 2:**
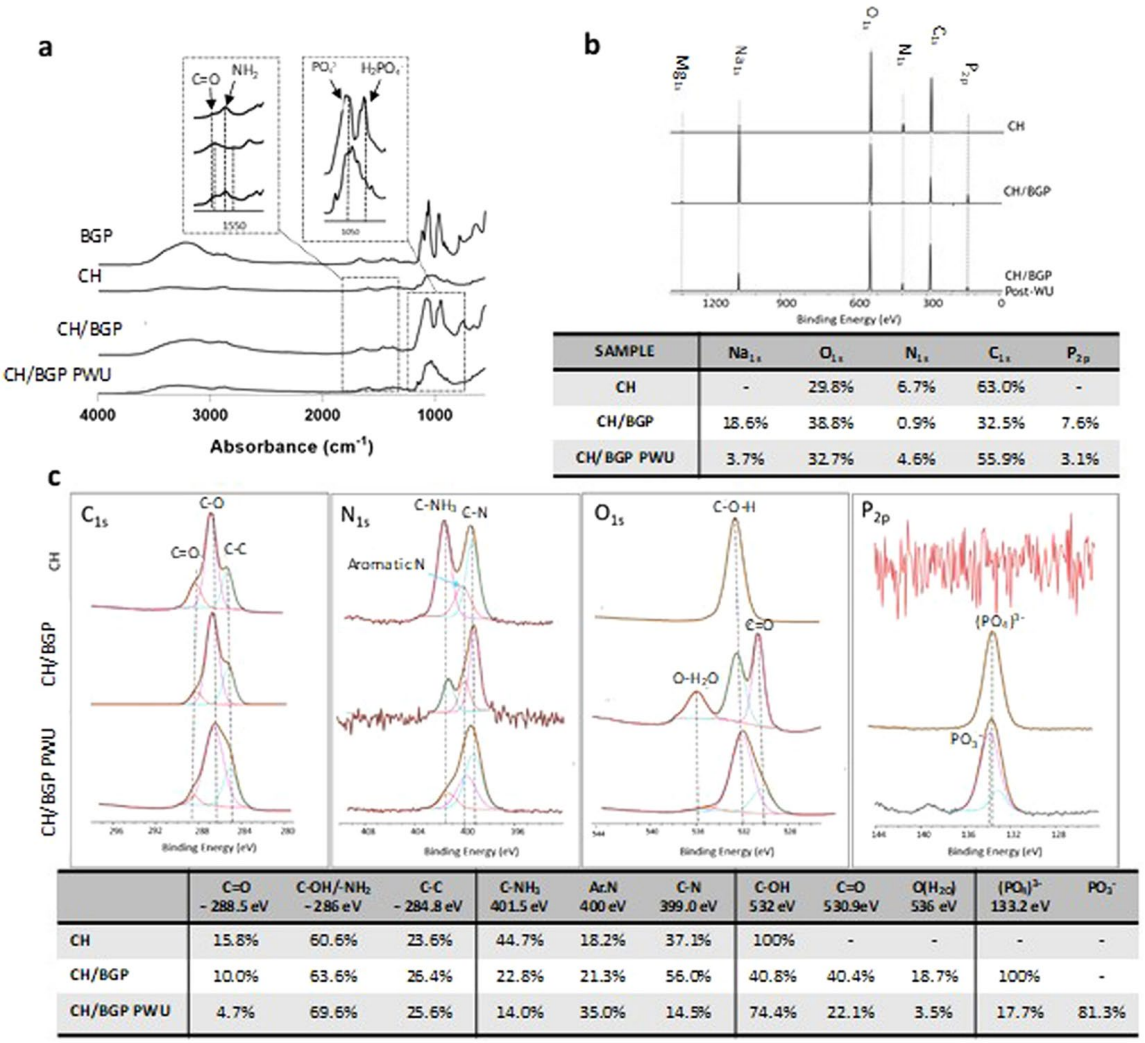
(**a**) FTIR-ATR spectra of BGP, CH, CH/BGP samples before and after water uptake in the range 4000–550 cm^−1^. (**b**) XPS spectra survey of BGP, CH, CH/BGP samples before and after water uptake and the relative atomic percentage of the main elements (Na_1s_, O_1s_, N_1s_, C_1s_, P_2p_). (**c**) XPS high resolution spectra, showing the deconvolution peaks deconvolution for CH and CH/BGP samples before and after water uptake (Post-WU).

Fourier Transformed Infrared analysis in Attenuated Total Reflectance (FTIR-ATR) spectra were measured for individual components (BGP and CH) as well as CH/BGP hydrogels before and after water uptake to analyse the chemical compositions (Fig. 2a**)**. BGP spectrum evidenced a small peak at 1078 cm^−1^, characteristic of C-O bond, and a sharpen peak at 963 cm^−1^, that was characteristic of P-O bonds. The fundamental peaks of CH spectrum were O-H and N-H overlapping band, observed in the range of 3600–3100 cm^−1^, the stretching vibration of aliphatic groups (-CH_2_ and -CH_3_), observed at 2970–2880 cm^−1^, primary and secondary amide bands (C=O and N-H) appearing at 1656 and 1601cm^−1^, peaks associated with OH oscillations and C-O stretching in the range of 1500 and 1200 cm^−1^ and, finally, the bridge (-O-) stretch of the glucosamine residues in the range 1200–800 cm^−1^ and a broad band at 660 cm^−1^ which was related with O=C-N vibrations. Compared to CH, the CH/BGP spectrum exhibited shift of the primary and secondary amide band respectively from 1656 to 1663cm^−1^ and from 1601 to 1567 cm^−1^ (Fig. 2a left insert) and a less intensity of C=O, O–H and N–H stretching peaks. Furthermore, two new chemical bands appeared at 1000 cm^−1^ and 800 cm^−1^ indicating aliphatic P-O-C stretching while the new band at 1050 cm^−1^ was characteristic of the -PO_4_^2−^ and a new shoulder at 920 cm^−1^ characteristic of the H_2_PO_4_^−^ (Fig. 2a right insert)^18^. After water uptake tests CH/BGP spectrum showed more similarities with the CH spectrum, where BGP characteristic peaks decreased in intensity. Figure 2b shows X-ray Photoelectron Spectroscopy (XPS) analysis reporting the survey spectra and relative atomic percentage of the main elements. The results showed the presence of O_1s_, nitrogen N_1s_ and carbon C_1s_ that were main characteristics of chitosan, in all the samples. The hydrogel formation led an evident decrease of N_1s_ and C_1s_ content from 6.7% to 0.9%, and from 63.0% to 32.5% respectively (CH vs CH/BGP). Furthermore Na_1s_ and P_2p_ peaks, BGP chemical elements, appeared after the hydrogel formation before (18.6% and 7.6%) and after water uptake (3.7% and 3.1%). The re-increase of those percentage (from 0.9% to 4.6% in N_1s_ and from 32.5% to 55.9% in C_1s_) was evident after water uptake. High resolution spectra (Fig. 2c) evidenced changes in bonds percentage, such as: C=O (288.5 eV) content decreased from 15.8% in CH to 10.0% and 4.7% respectively in CH/BGP before and after water uptake; while C-OH (532 eV) content decreased from 100% in the CH to 40.8% in CH/BGP and re-increased up to 74.4% after water uptake. New bonds appeared after the hydrogel formation: C=O (530.9 eV) with a content of 40.4% in CH/BGP and 22.1% after water uptake; O(H_2_O) (536 eV) with a 18.7% in CH/BGP and a decrease to 3.5% after water uptake; PO_4_^3−^ (133.2 eV) decreased up to 17.7% after water uptake. Finally, PO^3−^ (134 eV) was present in the CH/BGP samples after water uptake only.

### CH/BGP hydrogel morphology

**Figure 3:**
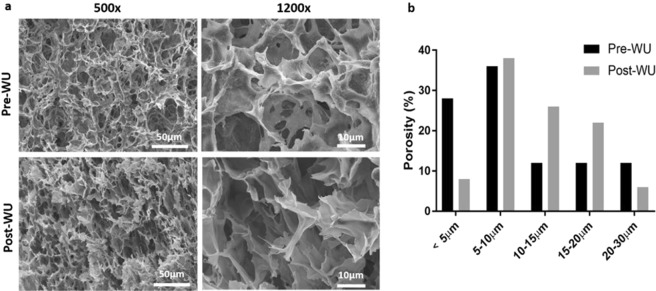
(**a**) ESEM images, representing cross-section microstructure of hydrogels before (Pre-WU) and post water-uptake (Post-WU) at 500x and 2000x magnification. (**b**) Distribution analysis of the pores within the ranges <5 μm, 5–10 μm, 10–15 μm, 15–20 μm and 20–30 μm.

Environmental scanning electron microscope (E-SEM) analyses were performed to evaluate the influence of BGP addition on the morphology of the freeze-dried samples (Fig. 3a). Both samples (before and after water uptake) showed a porous structure with “open-cell” structure, high degree of interconnectivity and irregular pores shape. However, after water uptake, samples evidenced smoother pore walls than as-prepared samples, more evident at the highest magnification (1200x). Furthermore, the water uptake influenced the pore size of the samples (Fig. 3b): pores with a diameter below 10 µm (64% of pores) with an average pore size measured of 5.6 ± 2.6 µm in the as-prepared samples, while after water uptake most of the pores were in the range 5–20 µm (86% of pores) with an average pore size of 11.5 ± 4.1 µm.

### Mechanical properties

Under static compression it was calculated a compressive elastic Young’s modulus of approximately 36 ± 4.0 kPa, while for the dynamic stress relaxation test an Equilibrium Young’s modulus of 17.4 ± 0.8 kPa was obtained after 1000 s. Furthermore, from the computational analysis done by using Matlab software of the stress-relaxation curves, the following relaxation times τ_1_ = 9.2 ± 0.5 s, τ_2_ = 65.0 ± 4.8 s and τ_3_ = 450.0 ± 35.7 s were calculated.

A further analysis on the mechanical properties has been performed by rheology. The linear viscoelastic region where storage modulus (G′) and the Loss modulus (G″) were independent from the shear strain was measured through strain sweep test, determining a linear behaviour for 0.1 to 10% strain. Then, the amplitude of strain for following rheological measurements was set at 1%. In the temperature sweep test, G′ and G″ slightly decreased with the increase in temperature until the crossing point where G′ > G″ (T = 32 ± 1 °C) and the sol/gel transition occurred. With the time sweep test, the time required for solution gelification at 37 °C (crossing point where G′ became higher than G″) was measured and the found gelation times was t_S/G_ = 144 ± 12 s. Figure S3 shows the graphs obtained from the mechanical analysis.

### Cytocompatibility evaluation

**Figure 4:**
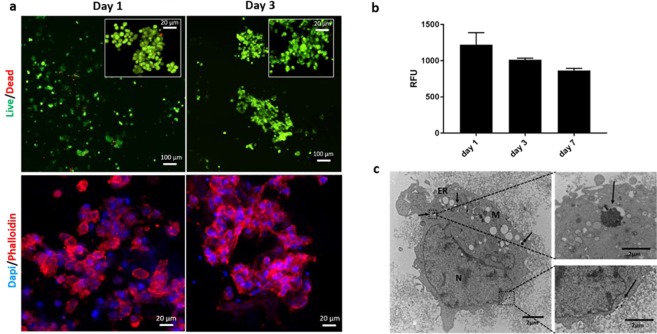
(**a**) Cytocompatibility evaluation of the MSCs-laden CH/BGP hydrogel: Live/Dead images of cells encapsulated in the CH/BGP hydrogel after 1d and 3d (green: live and red: dead cells) (top) and Immunostaining images of cells encapsulated in the CH/BGP hydrogel after 1d and 3d (blue: nuclei and red: cytoskeleton) (bottom). (**b**) PrestoBlue results for cells encapsulated in the hydrogel at 1d, 3d and 7d. RFU is referred to relative fluorescence units. No statistical differences were revealed. (**c**) TEM images of encapsulated MSCs in the hydrogel after 24 h incubation: black arrows indicates the salt uptake by cells, N labels nucleus, ER the endoplasmic reticulum and M mitochondria; in the inserts are shown magnifications of vacuole with salt (top) and endocytosis at cell surface process (bottom).

Live/dead staining showed that after 1 and 3 days in culture, the encapsulated MSCs into the hydrogels were viable and characterised by round-like morphology (Fig. 4a). Few dead (red) cells were observed. The immunostaining analysis (nuclei stained with 4′, 6- diamidino-2-phenylindole (DAPI) and the cytoskeleton with rhodamine-phalloidin) confirmed that the thermo-sensitive hydrogels were compatible with a tendency of the MSCs to form agglomerates after 3 days of culture. Cells metabolic activity resulted to be non-statistically different from day 1 to day 7 (1230 ± 150RFU to 800 ± 105RFU) (Fig. 4b), demonstrating that materials mixing and gelation process did not adversely affect the viability of the encapsulated cells. Finally, Transmission Electron Microscope (TEM) analysis evidenced that the structure of the cells were not altered after the cell culture in the gel (Fig. 4c): at the ultrastructural level, MSC showed large nucleus (N) with abundant chromatin, little marginally condensed heterochromatin with the presence of nucleoli; cell organelles (endoplasmic reticulum (ER) and mitochondria (M)) were not altered and a slight vacuolisation of the cytoplasm was observed. The arrows indicate the endocytosis process for the BGP salt internalisation with the formation of vacuoles.

### Neocartilage formation by MSCs-hACHs co-culture

**Figure 5:**
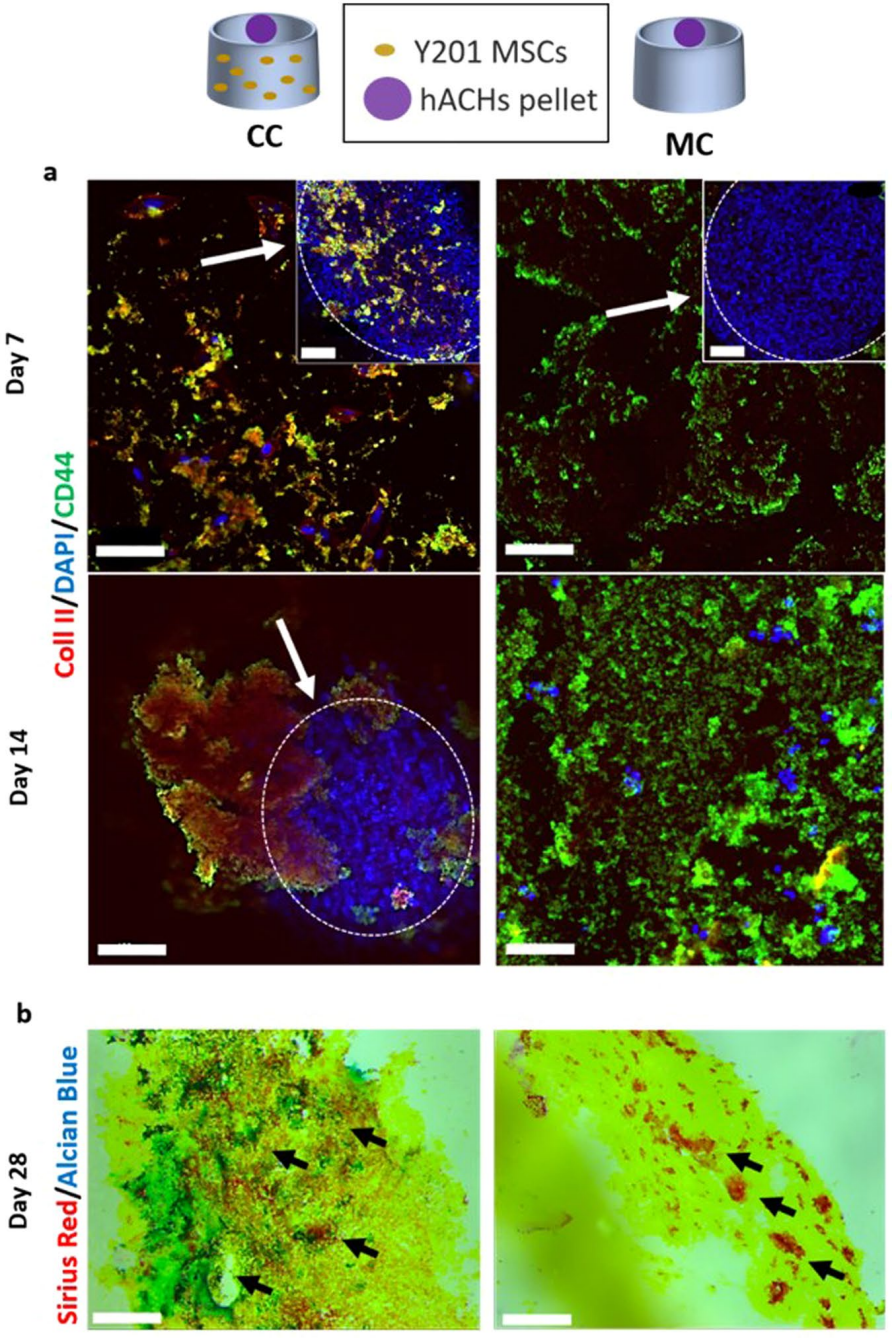
(**a**) Immunofluorescence staining at 7 (dashed line inserts represents spheroid area and white arrows are indicating at spheroids) and 14 days. Red staining for Col II, Blue for nuclei and Green for CD44 marker in co-culture (CC) (hACHs spheroid on MSCs-laden hydrogel) and mono-culture (MC) (hACHs spheroid on empty hydrogel). hACHs spheroids maintained their shape up to 14 days in the co-culture with MSCs while it disrupted after 7 days in the mono-culture due to the lack of MSCs stimuli. Bars = 100 µm. (**b**) Histological sections of the hydrogels after 28 culture days. GAGs were visualized by Alcian blue and Collagen by Sirius red staining respectively (Black arrows are indicating the area with higher production of collagen and GAGs). Bars = 200 µm.

Co-culture immunofluorescence analysis was performed after 7 and 14 days (Fig. 5a), staining cells nuclei in blue, Collagen Type II (Col II) fibres in red and CD44 in green. At day 7, the images report the hydrogel area around the spheroid and the spheroid itself. In co-culture condition, MSCs cells were homogenously dispersed within the gel while the chondrocyte spheroid was characterised by a round-like compact shape. A similar morphology of chondrocytes spheroid was found in the mono-culture, without chondrocytes migration into the hydrogel. CD44 was present within the spheroid and the hydrogel in the co-culture, and spread within the hydrogel in the mono-culture. Finally, in the co-culture conditions it was observed a small amount of Col II after 7 days of culture, while it was totally absent in the mono-culture. The results obtained on day 7 were compared with day 14. Particularly, in the co-culture, a higher production of Col II and a larger quantity of CD44 were evidenced compared to the mono-culture. For the co-culture 3D volumetric stack images up to 28 days were reported, showing clearly the Col II production increase over the time (Fig. S5). Histological analyses at 28days are shown in Fig. 5b.

After 4 weeks of co-culture of human Articular Chondrocytes (hACHs) spheroid on a MSCs-laden hydrogel in chondrocytes proliferation medium, the production of GAGs and collagen were clearly visible and homogenously distributed, compared to the hACHs mono-culture, where GAGs and collagen production was found to be low and fragmented. Furthermore comparing MSCs spheroid within the MSc-laden hydrogel in both chondrogenic and chondrocytes growth media, immunofluorescence analysis confirmed the increment of Col II, CD44 expression in the co-culture compared to mono-cultures (Fig. S6a) while histological analysis showed an enhancement of the collagen and GAGs accumulation when cells were in co-culture (Fig. S6b).

### Co-culture morphological analysis

**Figure 6:**
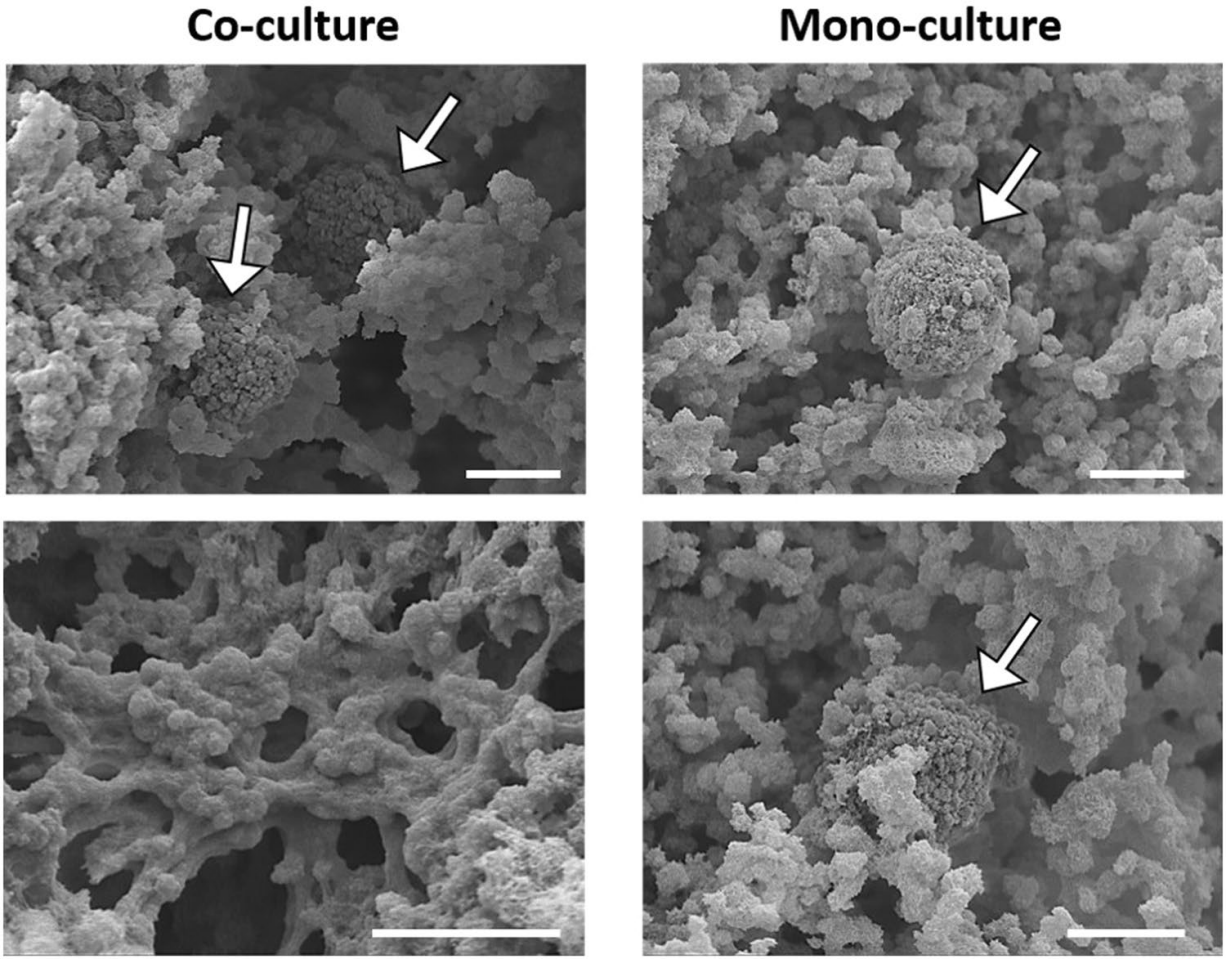
SEM micrographs of co-culture and mono-culture samples cross-sections at 45d post-culture. Black arrows indicate chondrocyte cells. Scale bars: 10 µm.

SEM investigation of the hydrogels surface and cross-sections revealed the presence of numerous cells entrapped within the matrix (Fig. 6). In co-culture condition chondrocytes cells, indicated by white arrows, were distributed within the pores of the matrix and exhibited a round-shaped morphology with a diameter <20 µm. A similar structure with round-shaped cells was found for the hACHs mono-culture. Finally, a fiber-like matrix dispersed within the cells in both conditions was observed (bottom images).

## Discussion

The CH/BGP formulation used in this work was optimised to obtain a gelation time of around 5 minutes at 37 °C (Fig. S2), suitable for future *in vivo* applications with a neutral pH (7.0–7.2) able to avoid possibility of unwanted effect on cell survival or tissue irritation at the injection site. Previous works have reported that a salt concentration increase leads to a gelation time decrease^19^. Specifically, at low BGP concentration the electrostatic repulsions between positively charged chitosan chains do not allow coagulation of chitosan chains and a minimum amount of BGP salt is needed to neutralise the positive charge density on chitosan chains^20^. Over this threshold, electrostatic attractions between the phosphate groups of BGP molecules and the amine groups of chitosan allow extensive hydrogen bonding via OH–NH and O–HN in the chitosan chains^15^. Furthermore other parameters may affect the gelation time, such as the chitosan DD (95% in this work), because the crosslink density increases between the phosphate groups and the amine groups^21^. A further key feature for injectable gels is the thermal stability of the formulation and the CH/BGP hydrogel showed a thermo-irreversible behaviour at RT and at 4 °C without changes in its physical state. This property, as demonstrated by Ganji *et al*., is related to BGP amount and the relative crosslinking strength, as well as to the competition between the different molecular forces involved in gel formation: in fact hydrogen bonds are not temperature dependent and, thus, cooling the hydrogels does not affect the gel physical structure^22,23^. The hydrogel was hydrophilic, with a high capacity to hold water molecules and to assure a good nutrient transport. The decrease in the water uptake percentage shown in Fig. 1 was correlated to the over-saturation concentration of the salt, fundamental for the obtainment of a fast gelification, as demonstrated by FTIR-ATR, with a reduction in BGP characteristic peaks and by XPS with significant decrease in Na_1s_ and P_2p_ intensity after WU tests^24^. SEM analysis showed that CH/BGP hydrogels possessed an open porous and interconnected network (more evident after WU due to the release of BGP excess). The hydrogels were characterised mainly by pores with diameters up to 30 µm, suitable for cell adhesion, nutrient absorption and diffusion (Fig. 3), and waste transport through the matrix^25–27^. In this work, the hydrogels showed a compressive Young’s modulus of around 40 kPa consistent with Kelly *et al*. that reported a compressive Young’s modulus ranging from 100–1000 kPa for native articular cartilage^28^. Other studies performed in literature found a compressive Young’s modulus with values ranging from 5 to 20 kPa^29,30^. Moreover, stress-relaxation behaviour of the prepared hydrogels was analysed, since it affects loads transfer and nutrients diffusion. The calculated relaxation times (τ_1_ = 9.2 ± 0.5 s, τ_2_ = 65.0 ± 4.8 s and τ_3_ = 450.0 ± 35.7 s), calculated following the generalised Maxwell model, consisting in three relaxation times (τ_1_ = 1–10 s, τ_2_ = 10–100 s and τ_3_ > 1000 s) were in the range of those reported in literature for polymeric gels by Wagenseil *et al*.^31^. These relaxation times are related with the equilibrium modulus, defined as the stiffness of the gel as all the fluids flows out, that was found ~17 kPa and are in accordance with those reported for native cartilage, which varies with depth from the articular surface and other similar studies^32,33^.

In evaluating MSC viability TEM images revealed intact nuclei, mitochondria, and endoplasmic reticula, together with the presence of endocytosis at the cell surface indicating internalization of the salt into the cell in forms of vacuoles (Fig. 4). From the Live/Dead and the PrestoBlue analyses, the vacuolisation process did not interfere negatively with cell viability and metabolic activity, potentially due to the consequent formation of a large autophagosome expelled through exocytosis^34^. These results are slightly in contrast with some works reported literature, where authors reported that cell viability was affected by BGP solution in a dose-dependent manner, due to a rapid release of BGP when immersed in medium, leading to hypertonicity of the medium^35^.

Last decade studies indicated that paracrine factors released by articular chondrocytes and their surface receptors were able to induce the chondrogenesis of MSCs, when in close-contact co-culture and deposit a cartilage specific matrix^23^. Therefore, in this work we designed a close-contact co-culture of hACHs spheroid on a MSCs-laden hydrogel for avoiding the chondrocytes issues of de-differentiation in fibroblasts when 2D cultured^36^. As shown in Fig. 5 the chondrocyte spheroid in co-culture maintained its shape at day 7 and day 14 in co-culture conditions while the chondrocyte spheroid in mono-culture disaggregated before 14 days, with the lack of stimulus from the MSCs considered to have led to the disaggregation. Furthermore, in the co-culture, many cells were observed in the proximity of the spheroid, rather than in other hydrogel areas and at 14 days all the nuclei appeared agglomerated in the spheroid, suggesting communication and migration of MSCs to the hACHs spheroid^37^. Moreover, immunofluorescence results confirmed that the interaction between chondrocytes and MSCs in the co-culture allowed a wide presence of CD44 marker after 7 and 14 days, both into and around the spheroid and Col II (increasing from day 1 to day 28 as shown in Fig. S5**)**, compared to the monoculture^38,39^. The benefit arising from close-contact co-culture was shown by histological analysis at 28 days, showing GAG accumulation and enhanced collagen production, uniformly distributed throughout the co-culture, with no evidence of this in the hACH mono-cultures (Fig. 6) or the MSC mono-cultures (Fig. S6b)^40^. After 45 days of culture round chondrocytes were uniformly distributed into the CH/BGP hydrogel in both the co-culture and mono-culture, residing in the cavities or by the edges of the cavities (Fig. 6). A thin fiber-like matrix can be observed, secreted by the cells with an effective interaction visible between cells and matrix^41^. Overall the model has shown that chondrocytes are positively influenced by MSCs in a co-culture, even when the MSCs are not in direct contact, and with initial signalling between cell types inhibited through gel encapsulation of the MSCs.

## Experimental Section

### Materials

Unless otherwise stated, reagents were obtained from Sigma-Aldrich, UK. The ultrapure water employed (dH_2_O) throughout the experiments was obtained with a Milli-Q Integral system equipped with a BioPak ultrafiltration cartridge (Millipore, Merck).

### Hydrogels preparation

A sterile 3.6% w/v chitosan solution (Mw = 100 kDa, DD 95%; HMC+, Germany) was prepared adding slowly CH to 0.2 M hydrochloric acid (HCl; reagent grade 37%) solution, autoclaved at 121 °C for 20 min, left under stirring until achieving achieve an homogenous solution at room temperature (RT) and, then stored at 5 °C. For the preparation of the thermo-sensitive hydrogel, 1104.5 mg of β-glycerophosphate disodium salt pentahydrate (Santa Cruz Biotec, US) were dissolved in 1 ml of sterile DPBS using a vortex (IKA, Germany) for few minutes; Dulbecco’s Modified Eagle’s Medium (DMEM) supplemented with 10% fetal bovine serum (FBS) and 5000 U/mL penicillin/streptomycin (P/S), was added to the salt solution in order to reach a final volume of 2.2 ml and then kept at 5 °C for 15 minutes. Finally, the prepared BGP solution was added drop-by-drop to the CH solution (5 ml) in an ice bath, under stirring to obtain a final concentration of 2.5%w/v. The CH/BGP solution was poured in 48-well plate and let it becoming gel at 37 °C in incubator (SANYO MCO-18M Multigas Incubator). Then gels were cut in disks (3–4 mm height x 6 mm diameter) for further characterisation and stored at 5 °C. A scheme of the hydrogel preparation and gelation process has been reported in Supplementary Data (Fig. S1).

### Gelation time and thermal irreversibility

Hydrogel gelation time was measured at three different temperatures (25, 37, 50 °C) using the test tube inverting method^19^. CH/BGP solutions (1 ml per bijou vial) were placed in a water bath to measure the gelation time at 37 and 50 °C or maintained at room temperature (RT) to measure it at 25 °C. The samples flowability was observed every 30 s by tilting the vials, considering the gelation time when the flow of the solution stopped. The same test was utilised to study the thermal reversibility of the gelation according the protocol proposed by Ganji *et al*.^22^. The obtained CH-based hydrogels were then cooled at a temperature of 4 °C or 25 °C. If the gels returned to a liquid solution, they were defined thermo-reversible hydrogels; otherwise the systems were considered thermo-irreversible.

### Water uptake analysis

For the water uptake (WU) analysis, hydrogels were frozen at −20 °C overnight and then lyophilised for 48 h in a freeze-dryer (Alpha 1–2 LDplus, CHRIST, Germany), weighted and placed separately in a 5 ml bijou vial containing 5 ml of DPBS and stored at 37 °C. The weight of all the samples was measured before immersion and after 30 min, 1, 2, 4, 6, 24 and 48 h of incubation. At each time, the samples were weighted after gently drying the extra DPBS on the surface using tissue papers. The water uptake percentage was calculated using the following equation:

1 $$WU( \% )=\frac{{W}_{t}-{W}_{i}}{{W}_{i}}\times 100$$

where W_i_ is the initial weight of the hydrogel and W_t_ is the weight of the hydrogel after the specific time point.

### Nutrients uptake and release

A glucose solution was prepared with the light sensitive 2-NBDG, dissolved in DPBS (0.06845 mg/ml) according the protocol proposed by Ribeiro-Conceicao *et al*.^42^. To test the glucose uptake a qualitative analysis was performed: 2 ml of CH/BGP solution was let to gelate in a 5 ml vial and then, 500 µl of 2-NBDG solution was added to the vial and observed over time. The time to obtain a complete yellow colour gel corresponded to the nutrients absorption time. For assessing the release over time, freeze dried samples were weighted, washed with DPBS and placed in a 48-well plate where 2 ml 2-NBDG solution was added to each gel during different time-points (30 min, 1, 3, 6, 24 and 48 h). At each time-point, gels were transferred to a new well-plate with DPBS to release the absorbed 2-NBDG. The released glucose solution was read at an excitation/emission of 465/540 nm using a LS-50B Luminescence Spectrometer (Perkin Elmer, Waltham, US).

### Morphological analysis

Freeze-dried hydrogels were investigated before and after water uptake analysis by E-SEM (XL30 FEG Philips) at accelerating voltage of 10 kV. The samples were cut into small squares (2 mm diameter x 1 mm height), fixed on the aluminium stub using carbon tape and gold-coated using a BIO-RAD Sputter Coater machine. For pore size evaluation E-SEM images were analysed using an image software (ImageJ). Three images for sample type were analysed measuring 50 pores for each one. The pore size was averaged to give a mean pore size assuming all pores were circular.

### Chemical characterisation

For the infrared spectroscopy CH and BGP raw powders and CH/BGP freeze-dried hydrogels (before and after water uptake) were analysed with a Spectrum Two PE instrument equipped with a horizontal attenuated total reflectance (ATR) crystal (ZnSe) (PerkinElmer Inc., US). Each spectrum was collected in absorbance mode as result of the average of 16 scans with 4 cm^−1^ resolution. Measurements were recorded in the wavelength range of 4000–550 cm^−1^.

Furthermore freeze dried hydrogels were examined by a scanning microprobe Kratos Axis Ultra-DLD XPS spectrometer (EPSRC service Cardiff, UK), equipped with a monochromatised AlKα X-ray radiation source. For each specimen, survey scans (Fixed Analyser Transmission mode, binding energy (BE) range 0–1200 eV, pass energy 117.4 eV) and high-resolution spectra were acquired of carbon (C_1s_), nitrogen (N_1s_), sodium (Na_1s_), oxygen (O_1s_), phosphorous (P_2p_). Atomic concentration (At.%) on the survey scan was performed using the built-in CasaXPS software package and in order to detect the BE representing the chemical binding states of the each elements within the films, the XPS spectra for the chemical elements detected from the films were subjected to peak deconvolution using the same software.

### Compression and stress-relaxation tests

Compression tests were performed using a universal testing machine (EZ-SX, Shimadzu, Japan) equipped with a 20 N load cell and crosshead speed set at 1 mm∙min^−1^. The compressive Young’s moduli (E) were calculated as the slope of the initial elastic region of the curve (0–10% strain). Then, stress-relaxation properties were evaluated following the protocol proposed by Bian *et al*., using a single compression ramp at a speed of 10% min^−1^ until reaching 10% strain^33^. Subsequently, the strain was held constant for 1000 s, while the load was recorded as a function of time. The equilibrium Young’s modulus (E_Y_) was determined by the equilibrium load obtained after 1000 s of relaxation under unconfined strain. The data obtained were analysed using MATLAB R2017 software^43^. By fitting a third order exponential decay to the relaxation curves, obeying the generalized Maxwell model, three relaxation times were acquired, as:

2 $$\sigma (t)=A{(e)}^{-t/{\tau }_{1}}+B{(e)}^{-t/{\tau }_{2}}+C{(e)}^{-t/{\tau }_{3}}+D$$

where A1, A2 and A3 were the amplitudes corresponding to the three different relaxation times τ1, τ2 and τ3.

### Rheological analysis

Rheological measurements were performed on an oscillatory and rotational Anton Paar’s Modular Compact Rheometer (MCR 302) using circular (diameter 5 cm) disks. The CH/BGP solution was prepared at 4 °C and poured between the two plates for the analysis. The Strain Sweep test was done to verify the values of the strain amplitude in order to ensure that all the measurements were performed within the linear viscoelastic region (were G′ and G″ were independent of the strain amplitude being linear and parallel between each other’s) at 37 °C with a rotational oscillation frequency of 1 Hz. The Temperature Sweep test was performed to determine the exact CH/BGP sol/gel transition temperature and this value is determined by G′ and G″ curves crossing. The oscillatory measurement was set at a frequency of 1 Hz, 1% strain amplitude (linear region of the strain sweep tests) and with a temperature rate of 5 °C/min in the range of 0–50 °C. The Time Sweep Test was done to know the exact sol/gel transition time and this value is known when G′ and G″ cross. To determine the gelation time, oscillatory measurements at 1 Hz and 1% strain, were started just after introducing cold solutions (at 4 °C) into the rheometer chamber pre-heated at 37 °C. The temperature was constant during the test at the same value.

### Cells culture and encapsulation protocol

Human TERT immortalised bone marrow stromal cell line was kindly supplied by Prof P. Genever (York University) at passage 84 and cultured as reported^44^. Briefly, cells were grown at 37 °C, 5% CO_2_, in DMEM with low glucose content, supplemented with 10% FBS, 2 mM L-glutamine and a 1% P/S. After the expansion, cells were used between passage 86 and 90. CH/BGP solution (200 µL) was poured into a membrane-based cell culture insert (membrane pore size of 8.0μm, Merck, Millipore, Germany) at room temperature and Y201 MSCs were added and mixed gently with the CH/BGP solution at an optimised cellular density of 2 × 10^6^ cell ml^−1^ following the consideration reported also by Liu *et al*.^45^. Samples were placed in 24-well plates and allowed to gelate in the incubator (37 °C and 5% CO_2_). Finally, after 30 min, 1 ml of fresh DMEM was added into each well and refreshed three times per week. Human articular chondrocytes (hACHs) were cultured as recommended by the seller. Cells were cultured in Chondrocyte Growth Medium ready-to-use (PromoCell, UK) at 37 °C in a humidified atmosphere incubator containing 5% CO_2_ and sub-cultured to passage 5–6 for the experiments.

### Co-culture assessment

To set up a direct-contact co-culture system, a chondrocyte spheroid was seeded on the MSCs-laden hydrogel, supplemented with chondrocytes growth media and compared with a mono-culture made of a chondrocytes spheroid seeded on plain hydrogel (without MSCs). For spheroids formation, hACHs dispersed in chondrocytes growth medium supplemented with 0.25% Methylcellulose were seeded in a round bottom 96-wells plate (non-tissue culture treated) at a density of 2 × 10^5^ cells/well^44,46^. After one day, the hydrogels were prepared following the same protocol, but using 100 µL of solution with the same MSCs cell density (2 × 10^6^ cell ml^−1^). After formation of the hydrogels, spheroids were aspired from the 96-well plate and placed on the top of the respective gels and the respective media were added. Further controls of MSCs mono-cultures, supplemented with chondrogenic media or chondrocytes growth media were analysed (reported in the Supporting Information).

### Cytotoxicity assay

TEM (Philips CM 100 Compustage FEI) was used for investigating the polymer localization and cell structure (organelles and internal structure) at voltage of 100 kV. Digital images were collected using an AMT CCD camera (Deben). Prior to the analysis, cells encapsulated in the hydrogels were fixed overnight using a pre-warmed solution of 2% glutaraldehyde (TAAB Laboratory Equipment) in sodium cacodylate buffer at 4 °C, followed by a post-fixation with 1% osmium tetroxide (Agar Scientific). After various dehydration steps, hydrogels were embedded in resin, and cut in ultrathin sections using a diamond knife on a Leica EM UC7 ultra microtome (Leica Microsystems). The sections were stretched with chloroform to eliminate compression, mounted on Pioloform-filmed copper grids (Agar Scientific) and ready to be visualised. Further cytotoxicity assessment are reported in Supporting Information.

### Hydrogel cytocompatibility

Live/Dead assay (LIVE/DEAD® Cell Imaging Kit, Life Technologies, UK) was used according to the manufacturer’s instructions. This fluorescence-based kit combines calcein AM and ethidium bromide to yield two-colour discrimination of the population of live cells (green) from the dead cells (red). Each cell culture condition was washed twice with PBS before incubation with staining. In brief, 4 μM ethidium homodimer-1 and 10 μM calcein dilute in DPBS, were incubated with the cell-encapsulated samples for 30 min at 37 °C^47^. Images were collected at 1 and 3 days using a Nikon A1R inverted confocal microscope^48^.

To test the cells metabolic activity, culture medium was removed at each time point (1, 3 and 7 days), samples were washed with DPBS and 1 ml of PrestoBlue™ reagent (Thermo Scientific, UK) diluted in DMEM (1:10) protected from light; was added to each well with the gel and incubated for 2.5 h at 37 °C and 5% CO_2_. Then, 200 µl of each well solution (in triplicate) was transferred to a white bottom 96-well plate and a LS-50B Luminescence Spectrometer (Perkin Elmer, Waltham, MA) was used to measure the fluorescence (excitation/emission of 560/590 nm). Then, samples were washed with PBS twice and fresh media was added for the next time point.

For immunostaining analysis, samples were fixed in pre-warmed 4% w/v paraformaldehyde (PFA) and cells were consequently permeabilised using 0.1% v/v Tween20® in DPBS for three washes. Rhodamine-phalloidin was prepared using phalloidin-tetramethylrhodamine B isothiocyanate (1:1000 in 0.1% DPBS/Tween20®) for 20 min at RT. Then, samples were washed with 0.1% DPBS/Tween20® solution and immersed in DAPI solution (Vector Laboratories,UK) (1:2500 in 0.1% DPBS/Tween20®) for 10 min at RT. Experiments were light sensitive and images were collected at 1 and 3 days using a Nikon A1R inverted confocal microscope.

### Co-culture immunofluorescence analysis

Co-culture samples were fixed as for the immunostaining, blocked for 1 h in DPBS supplemented with 2% BSA at 4 °C and incubated overnight with primary antibodies: polyclonal Anti-Collagen II (Anti-Col II; ab34712, abcam) diluted in 0.1% DPBS/Tween20® (1:200) and monoclonal Anti-CD44 (ab189524, abcam) diluted in 0.1% DPBS/Tween20® (1:250), following the product datasheets^49^. Samples were washed twice with 0.1% DPBS/Tween20® for 5 min and incubated with Anti-Col II secondary antibody, Alexa Fluor® goat anti-rabbit igG (H + L) (ab150080, abcam) diluted in 0.1% DPBS/Tween20® (1:1000) for 1 h at RT. Then, samples were washed twice with 0.1% DPBS/Tween20® for 5 min and incubated with Anti-CD44 secondary fluorescein-labelled goat anti-rabbit IgG (H + L) (F2765, Thermo Fisher Scientific) diluted in 0.1% DPBS/Tween20® (1:1000), for 1 h at RT. Sample were re-washed twice with 0.1% DPBS/Tween20® and DAPI staining for nuclei was performed (as explained before) and imaged using a Nikon A1R inverted confocal microscope at 7 and 14 days.

### Co-culture histological analysis

Co-culture samples were fixed in pre-warmed 10% formalin (24 h), and transferred to 70% Ethanol (EtOH) (Thermo Fisher Scientific). Then, samples were transferred to histology cassettes lined with biopsy sponges and dehydrate in graded ethanol series (80%, 95% and 100% EtOH) for 30 min each, followed by two clarification steps in xylene (Thermo Fisher Scientific)^50^. Following this, samples were paraffin-embedded following routine histological procedures and sectioned in three 5µm-thick slices. The slices were fixed on glass slides, deparaffined and then stained for the collagen and GAGs production analysis. In order to evaluate GAGs production, Alcian Blue acid mucins staining was done. Slides were washed in running water and stained for 20 min with Alcian Blue solution (pH 5), prepared by dissolving 5 mg of Alcian Blue 8GX (Merck) in 500 ml dH_2_O. Then, slides were rinsed in running dH_2_O. The amount of collagen deposited by the cells was investigated using the Sirius Red assay^51^. Sections, were washed in two changes of acidified water, made diluting 0.5 ml of glacial acetic acid in 100 ml of dH_2_O. Then, samples were treated with Sirius-red/picric acid solution (Sircol™, Biocolor Ltd., UK) for 1 h at room temperature and re-washed in two changes of acidified water. Slides underwent a dehydration process in three rapid changes of absolute alcohol and cleared in Xylene and mounted in DPX Mounting for histology. Stained slides were covered with a glass slide and imaged with Leica Stereomicroscope (Bright field) and analysed with Leica software.

### Co-culture morphological analysis

The morphology of the cells within the scaffolds were observed using SEM after 28days of co-culture. Samples were fixed in pre-warmed 2% Glutaraldehyde overnight, rinsed in PBS twice and dehydrated in ethanol grades: 30 min in each 25%, 50% and 75% EtOH and 1 h in 100% EtOH (twice). Samples were stored at 4 °C in 100% EtOH until critical point dried using a BAL-TEC 030 Critical Point Dryer (Leica Geosystems Ltd, Milton Keynes, UK). Finally, gels were mounted on carbon discs (TAAB Laboratory Equipment) and gold-coated using a Polaron E5000 SEM Coating unit (Quorum Technologies Ltd, UK). After gold coating using Polaron SEM coating unit with 15 nm of gold samples were imaged at different magnifications. SEM images for MSCs controls are reported in the Supporting Informations (Fig. S6).

### Statistical analysis

Tests were performed at least in triplicate for each sample. The results were represented as mean ± standard deviation. Differences between groups were determined using One-way analysis of variance (ANOVA) with Turkey’s multiple comparison test using levels of statistical significance of p < 0.05(*).

## Conclusion

The fabricated thermo-sensitive chitosan-based hydrogels presented fast sol/gel transition time that allows cell encapsulation, an intrinsic porous structure, and a high fluid uptake capability. The gel based co-culture system allows for the influence of two cell types on one another to be investigated with the gel offering tissue-like diffusion rates of cell signalling molecules, and showed that encapsulated MSCs stimulate chondrocyte activity within a gel co-culture, both in terms of maintaining the coherence of chondrocyte spheroids and leading to enhanced collagen production. A further investigation could be the assessment of the hydrogel system as *in vitr*o model for mimicking native cartilage tissue for e.g. studying pathological processes or drug screening or as *in vivo* carrier in order to transplant the chondrocytes spheroid to the target site immediately after formation via surface entrapment onto the CH/BGP hydrogel.

## Supplementary information

Supplementary information

## Data Availability

The datasets generated and/or analysed during the current study are available from the corresponding author on reasonable request. See Supplementary Information for cytotoxicity tests on BGP, representative stress-strain curves and rheology plots on CH/BGP hydrogels, and biological assessment of the monoculture of Y201 MSCs.
