# Supplementary material for: The interplay between chondrocyte spheroids and mesenchymal stem cells boosts cartilage regeneration within a 3D natural-based hydrogel
Source: Sci Rep. 2019 Oct 10;9:14630. doi: 10.1038/s41598-019-51070-7 (PMC6787336; doi:10.1038/s41598-019-51070-7)
Supplement: Supplementary file 1 — Supplementary information [file 41598_2019_51070_MOESM1_ESM.pdf]

The interplay between chondrocyte spheroids and mesenchymal stem cells boots cartilage regeneration within a 3D natural-based hydrogel

Annachiara Scalzone[1], Ana M. Ferreira[1], Chiara Tonda-Turo[2], Gianluca Ciardelli[2], Kenny Dalgarno[1], Piergiorgio Gentile[1*]

[1]School of Engineering, Newcastle University, Claremont Road, Newcastle upon Tyne NE1 7RU, United Kingdom

[2]Department of Mechanical and Aerospace Engineering (DIMEAS), Politecnico di Torino Corso Duca degli Abruzzi 29, Turin 10129, Italy

Corresponding author e-mail: *piergiorgio.gentile@ncl.ac.uk

Supporting data

Cytotoxicity of the gel

Hydrogel cytotoxicity was evaluated on hydrogel extracts following the international standard ISO 10993. Briefly, 0.5ml of CH/BGP solution was poured in a 12-well plate and let gellify at 37°C in an incubator. Then 2.5ml DMEM (supplemented with FBS and P/S) was added on the top of each gel and incubated for 24h. Y201 cells were seeded in 96-well plate at a density of 5000cells/well and in a 48-well plate (on 6 mm diameter coverslips) at a density of 30000 cells/well and incubated for 12h. Then, the culture medium was removed and replaced with 100μl hydrogel extracts. Cytotoxicity has been evaluated after 24h and 48h through live/dead on 48-well plate seeded cells coverslip and PrestoBlue on 96-well plate seeded cells, performed as explained in the main text.

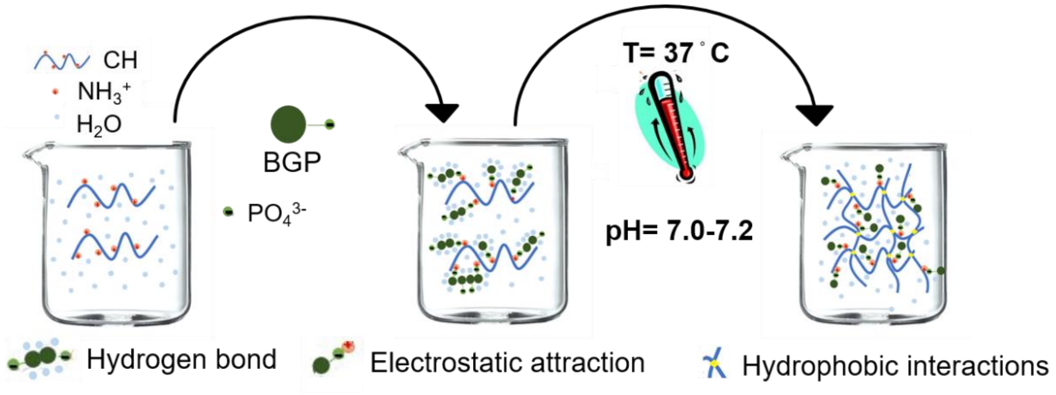

Figure S1: Scheme of the Hydrogel gelation process. CH (3.6%w/v) was dissolved in 5 ml of 0.2M HCl and BGP (1.1g in 2.2 ml of PBS) was added to obtain a 2.5% w/v solution for sol/gel transition at 37°C and pH 7.4.

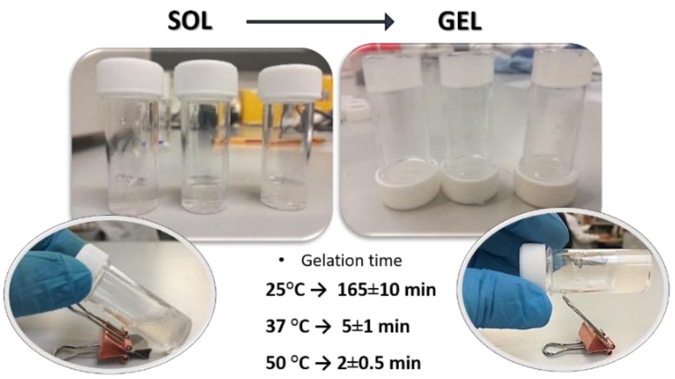

Figure S2: Sol/gel transition of CH/BGP with temperature. It is shown the tube inverted method in the images (a triplicate for each temperature) and it is reported the gelation time at the 5, 25 and 37°C on the bottom.

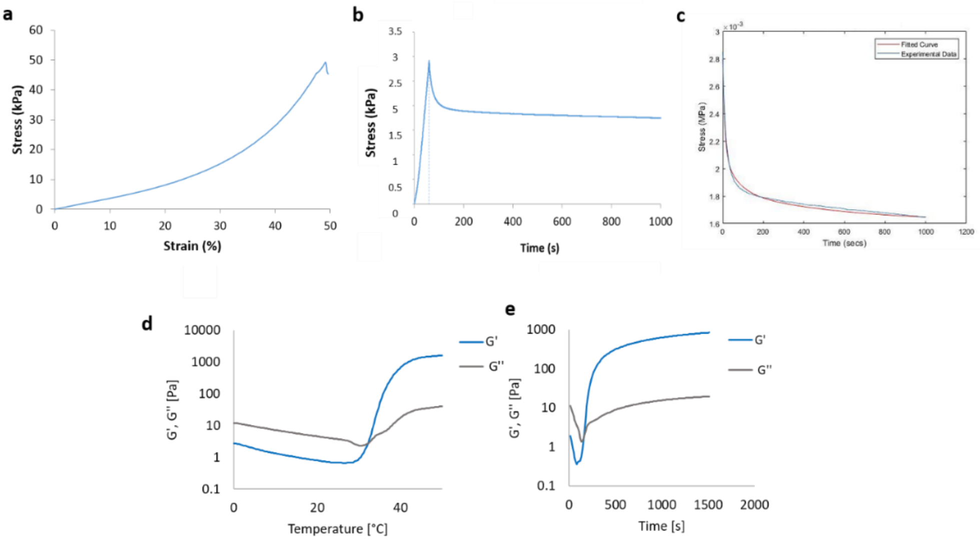

Figure S3: Mechanical analysis graphs. (a) Compression test. (b) Stress-relaxation curve from experimental data. (c) The typical stress relaxation curve obtained after 1000 s, fitting the experimental data with a third order exponential. (d) Temperature sweep test rheological analysis. (e) Time sweep test rheological analysis.

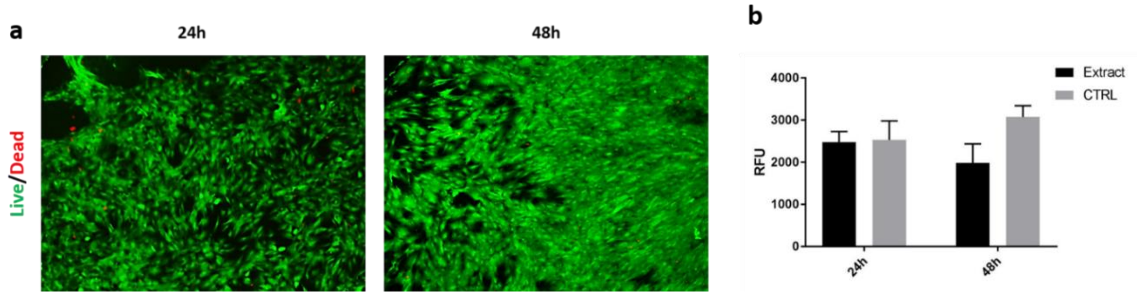

Figure S4: Cytotoxicity assay results. (a) Live/Dead staining images of MSCs incubated with the gel extract at 24h and 48h (Green for live cells and red for dead cells).(b) Metabolic activity of the MSCs incubated with the gel extract or with the normal media as control, at 24 and 48h. RFU is referred to relative fluorescence units. No statistical differences were found.

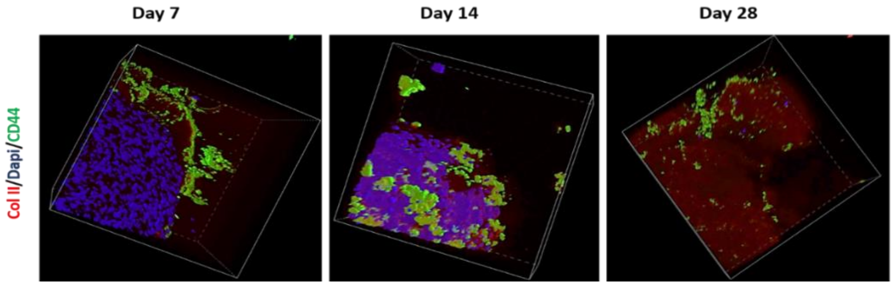

Figure S5: Confocal microscopy volume stack of the co-culture. Staining: Cells nuclei in the gel (DAPI, blue), collagen II (red) and CD44-chondrogenic marker (green) at three time points (7, 14 and 28 days).

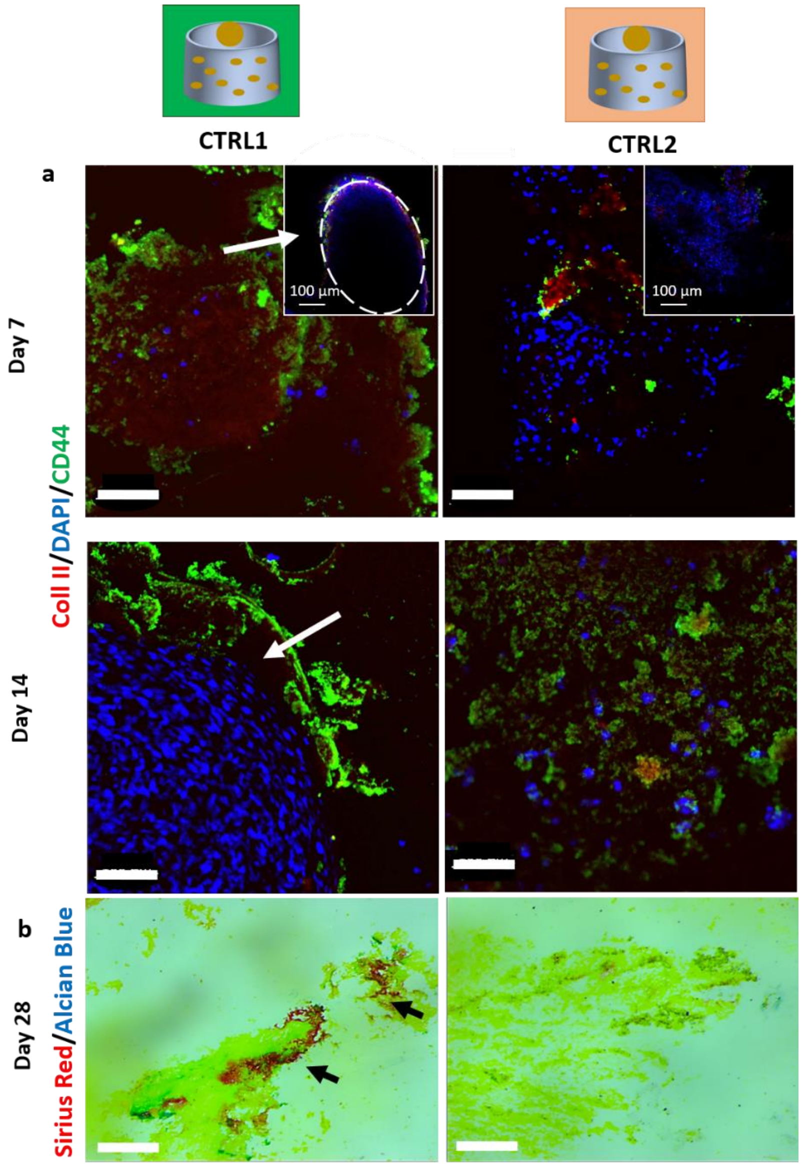

Figure S6: **(a)** Immunofluorescence staining at 7 and 14 days. Red staining for Col II, Blue for nuclei and Green for CD44 marker at two different culture conditions: CTRL1 (MSCs spheroid on MSCs-laden hydrogel in chondrogenic media), CTRL2 (MSCs spheroid on MSCs-laden hydrogel in chondrocytes growth media) Bars=100µm. **(b)** Histological sections of the hydrogels after 28 culture days. GAGs were visualized by Alcian blue and Collagen by Sirius red staining respectively. Bars=200µm

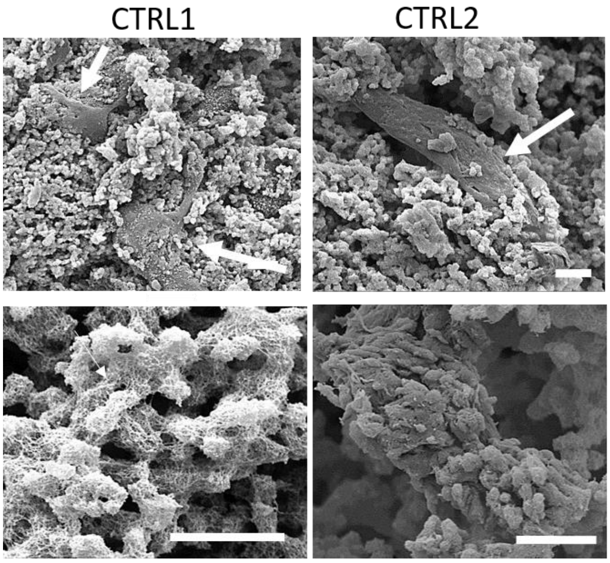

Figure S7: SEM micrographs of control cross-sections at 45 days post-culture. (CTRL1 (MSCs spheroid on MSCs-laden hydrogel in chondrogenic media), CTRL2 (MSCs spheroid on MSCs-laden hydrogel in chondrocytes growth media)). White arrows are indicating MSCs cells. Scale bars:10µm.
